# Potential Role of Adult Stem Cells in Obstructive Sleep Apnea

**DOI:** 10.3389/fneur.2012.00112

**Published:** 2012-07-11

**Authors:** Isaac Almendros, Alba Carreras, Josep M. Montserrat, David Gozal, Daniel Navajas, Ramon Farre

**Affiliations:** ^1^CIBER Enfermedades RespiratoriasBunyola, Spain; ^2^Department of Pediatrics, Pritzker School of Medicine, Comer Children’s Hospital, The University of ChicagoChicago, IL, USA; ^3^Sleep Lab, Hospital Clínic BarcelonaBarcelona, Spain; ^4^Institut Investigacions Biomediques August Pi SunyerBarcelona, Spain; ^5^Unitat de Biofisica I Bioenginyeria, Universitat de BarcelonaBarcelona, Spain; ^6^Institut de Bioenyingeria de CatalunyaBarcelona, Spain

**Keywords:** sleep apnea, endothelial progenitor cells, very small-like embryonic stem cells, mesenchymal stem cells

## Abstract

Adult stem cells are undifferentiated cells that can be mobilized from the bone marrow or other organs, home into injured tissues, and differentiate into different cell phenotypes to serve in a repairing capacity. Furthermore, these cells can respond to inflammation and oxidative stress by exhibiting immunomodulatory properties. The protective and reparative roles of mesenchymal stem cells (MSCs), very small embryonic-like stem cells (VSELs), and endothelial progenitor cells (EPCs) have primarily been examined and characterized in auto-immune and cardiovascular diseases. Obstructive sleep apnea (OSA) is a very prevalent disease (4–5% of adult population and 2–3% of children) characterized by an abnormal increase in upper airway collapsibility. Recurrent airway obstructions elicit arterial oxygen desaturations, increased inspiratory efforts, and sleep fragmentation, which have been associated with important long-term neurocognitive, metabolic, and cardiovascular consequences. Since inflammation, oxidative stress and endothelial dysfunction are key factors in the development of the morbid consequences of OSA, bone marrow-derived stem cells could be important modulators of the morbid phenotype by affording a protective role. This mini-review is focused on the recent data available on EPCs, VSELs, and MSCs in both animal models and patients with OSA.

## Adult Bone-Marrow Derived Stem Cells and Obstructive Sleep Apnea

Adult stem cells are undifferentiated cells that are present in most adult tissues and more particularly in the bone marrow. Several stimuli, such as inflammation and oxidative stress, can mobilize these cells from the bone marrow into the circulating blood, attract them to the injured site(s) and home them into these tissues to enable tissue repair and regeneration, since adult stem cells can differentiate into specialized cell types of an organ or tissue (Tousoulis et al., 2008; Zuba-Surma et al., 2011). Whereas some types of bone marrow-derived stem cells are relatively well characterized – e.g., mesenchymal stem cells (MSCs) –, other stem cell types, such as endothelial progenitor cells (EPCs) and very small embryonic-like stem cells (VSELs), have been discovered more recently, and their characterization is not as comprehensive. A salient finding from a very recent investigation on adult stem cells was that these cells – in addition to their well-known reparative functions resulting from differentiation into tissue injured phenotypes – also participate in the physiological responses to inflammation and other injurious challenges. The increased understanding of the role of bone marrow-derived stem cells in tissue and organ homeostasis has boosted research into their potential applications in cell-therapy approaches to different diseases, particularly those in which ischemia-reperfusion plays a central role.

There is increasing evidence that obstructive sleep apnea (OSA) is associated with several important cardiovascular consequences, such as hypertension, myocardial infarction and angina pectoris, arrhythmias, and stroke (Jelic and Le Jemtel, 2008; Bradley and Floras, 2009; Kohler and Stradling, 2010; Lavie, 2012). Most of these consequences are attributed to a common pathway, namely, accelerated atherogenesis (Drager et al., 2005; Jelic and Le Jemtel, 2008; Chung et al., 2009; Kohler and Stradling, 2010; Quercioli et al., 2010; Lurie, 2011). In fact, OSA can promote impaired vasodilation (Farre et al., 2007; Nacher et al., 2009; Reichmuth et al., 2009; Lavie, 2012), expression of pro-inflammatory and prothrombotic factors (Nacher et al., 2007; von Kanel et al., 2007; Gozal et al., 2008; Jelic and Le Jemtel, 2008; Lurie, 2011; Lavie, 2012), all of which can contribute to the endothelial injury and vascular dysfunction associated with this highly prevalent condition. The cumulative evidence derived from extensive clinical and translational research carried out in this field suggests that intermittent hypoxia (IH), increased inspiratory efforts, and recurrent arousals from sleep are the main determinants underlying the inflammatory response and cardiovascular alterations observed in OSA (Nacher et al., 2009; Kohler and Stradling, 2010; Almendros et al., 2011).

In the context of the primary mechanisms involved in the inflammation and oxidative stress that characterize OSA, a striking similarity to the mechanisms underlying ischemia-reperfusion injury becomes immediately apparent. Accordingly, the corollary hypothesis that adult stem cells could play a role in the pathophysiology of OSA and its associated end-organ morbidities would not be farfetched, considering the known role that stem cells play in ischemia-reperfusion injury. Therefore, it has also been suggested that bone marrow-derived stem cells may participate in affording protection in patients with OSA. Indeed, recent studies carried out on both animal models and patients have provided exciting initial evidence to support the notion that adult stem cells may indeed play a homeostatic role in the inflammatory and endothelial dysfunction processes of OSA (Carreras et al., 2009, 2010a,b; Jelic et al., 2009; Kheirandish-Gozal et al., 2010).

## Endothelial Progenitor Cells in OSA

Endothelial progenitor cells (EPCs) are adult bone marrow-derived stem cells involved in endothelial repair through re-endothelization of injured vessels and neovascularization of ischemic lesions. These cells, which can be isolated from several reservoirs, such as circulating peripheral blood (Asahara et al., 1997), bone marrow (Reyes et al., 2002), and cord blood (Murohara et al., 2000), represent a pool of cells that contributes to endothelial repair (Hill et al., 2003; Zampetaki et al., 2008). Moreover, cardiovascular risk has been correlated with reduced functionality (Vasa et al., 2001; Hill et al., 2003) and less numbers of circulating EPCs determined as CD34/KDR double-positive cells (Vasa et al., 2001), and conversely, an increased EPCs functionality has been reported as predictive of beneficial responses in different situations associated with cardiovascular disease events (Hill et al., 2003; Werner et al., 2005).

Studies performed in animal models have shown that a selected number of cytokines (Takahashi et al., 1999; Amano et al., 2004; Tousoulis et al., 2008), vascular endothelial growth factor (VEGF; Gill et al., 2001; Amano et al., 2004), and oxidative stress (Thum et al., 2006; Tousoulis et al., 2008) are all able to mobilize EPCs from the bone marrow. In the clinical setting, there is increasing evidence that chronic inflammation and oxidative stress may induce EPCs dysfunction (Tousoulis et al., 2008). Therefore, the putative protective role of these cells and their dysfunction in some diseases open potential new therapeutic venues. For example, transplantation of bone marrow-derived EPCs reduced infarct volume and neurological deficits after acute focal brain ischemia-reperfusion injury (Ohta et al., 2006). Moreover, clinical studies carried out to date endorse the potential of therapy based on transplantation of EPCs for several diseases, including myocardial infarction and ischemic stroke (Bolli et al., 2011; Borlongan et al., 2011).

Among the studies carried out on EPCs in OSA, these have been carried out in both children and adult patients – and in most cases co-morbidities involving a potential cardiovascular risk – such as obesity – have been avoided. However, the cumulative results currently available on the role of EPCs in OSA are controversial, probably because these studies were performed on only a small number of participants (Table 1) and/or the different markers used to characterize these cells (Berger and Lavie, 2011).

**Table 1:** **Bone marrow-derived stem cell studies in patients with obstructive sleep apnea (OSA) and animal models**.

Reference	Species	Groups (*n*)	Main results
**EPCs**			
Kizawa et al. (2009)	Human	Control (38) OSA (37)	EPCs were increased threefold in OSA patients with respect to controls and decreased after 12 weeks of CPAP treatment.
Martin et al. (2008)	Human	Control (10) OSA (17)	EPCs: no changes between OSA and control groups.
Yun et al. (2010)	Human	Control (22) OSA (82)	EPCs: similar values in both groups. Endothelial impairment in OSA group.
de la Peña et al. (2008)	Human	Control (13) OSA (13)	EPCs from OSA were reduced fivefold with respect to control group. OSA also presented increased levels of VEGF but endothelial function was unaltered.
Jelic et al. (2008)	Human	Control (15) OSA (30)	OSA group presented a fourfold reduction in circulating EPCs with respect to controls. Levels were normalized after 4 weeks of CPAP. In addition, OSA patients presented increased levels of oxidative stress and inflammation.
Jelic et al. (2009)	Human	Control (16) OSA (16)	EPCs were reduced threefold with respect to controls and were inversely related to the presence of endothelial microparticles. EPCs increased after 4 weeks of CPAP treatment.
Murri et al. (2011)	Human	OSA (16)	Negative correlation of circulating EPCs with severity of OSA and oxidative stress. EPCs values returned to control values after 1 month of CPAP treatment.
Kheirandish-Gozal et al. (2010)	Human	Control (20) OSA (40)	Circulating EPCs were reduced in those OSA children with impaired endothelial function (20 patients) and increased in those without it (20 patients).
**VSELs**			
Gharib et al. (2010)	Mice	Control (30) (21% O_2_ for 48 h) Int. hyp. (30) (21.0 and 5.7% O_2_ every 180 s 12 h/day for 48 h.	Intermittent hypoxia (IH) induced migration of VSELs from bone marrow to peripheral blood. More than 1,100 genes were differentially expressed in VSELs in response to IH.
Gharib et al. (2011)	Mice	Control (6) (21% FiO_2_ for 24 h) Chr. Hyp. (6) (8% FiO_2_ for 24 h)	Hypoxia mobilized VSELs from the bone marrow to peripheral blood and induced a distinct genome-wide transcriptional signature.
**MSCs**			
Carreras et al. (2009)	Rat	Control (10) OSA (10) (60 h 15 s each) for 5 hours	Circulating MSCs were three times higher in rats subjected to OSA than in controls.
Carreras et al. (2010a)	Rat	Cont. (30) OSA (30) (60 h 15 s each) for 5 h	Serum from apneic rats increased MSCs migration, adhesion and endothelial wound healing compared to serum from control rats.
Carreras et al. (2010b)	Rat	Cont. (10) OSA (10) (60 h 15 s each) for 5 h	IL-1α was higher in rats subjected to recurrent obstructive apneas than in controls. MSCs injection reduced the IL-1α levels induced by recurrent obstructive apneas.

*EPCs, endothelial progenitor cells; MSCs, mesenchymal stem cells; VSELs, very small embryonic-like cells*.

For example, in adult patients with OSA, EPCs have either been reported as increased, decreased or have remained unchanged; in most studies however, a reduction in circulating EPCs has emerged. Moreover, these studies suggest a potential role for EPCs in vascular repair. Kizawa et al. (2009) reported a ~threefold increase in EPCs in OSA, which was reverted after treatment with continuous positive airway pressure (CPAP). Martin et al. (2008) found similar values of EPCs in OSA patients and controls. Yun et al. (2010) did not find any differences in EPCs in OSA patients when compared to healthy subjects, but reported an increase in endothelial microparticles (marker of endothelial damage) that correlated with the apnea-hypopnea index. In contrast with these findings, data from five recent studies reported a decrease in EPCs in OSA patients. Jelic et al. (2008, 2009) showed that the number of circulating EPCs in OSA patients (newly diagnosed with AHI ≥ 5 and BMI < 30) was reduced when compared to age-matched controls and that after 4 weeks of CPAP treatment (adherence ≥ 4 h/day), the number of circulating EPC either normalized (Jelic et al., 2009) or even increased (Jelic et al., 2008) compared to controls. Furthermore, a role of EPCs in endothelial repair capacity in OSA was suggested by the negative correlation between circulating EPCs and endothelial apoptotic microparticles (Jelic et al., 2009). Similarly, Murri et al. (2011) reported a reduction in circulating endothelial-specific progenitor cell markers and a negative correlation with the severity of OSA and of oxidative stress. The values of circulating endothelial-specific progenitor cell markers also returned to control values after 1 month of CPAP treatment. In another study, de la Peña et al. (2008) studied OSA patients free of any other known cardiovascular risk factors, and reported a reduced number of circulating EPCs, as well as an increase in plasma VEGF levels compared to age- and sex-matched controls.

The role on EPCs in children with OSA remains virtually unexplored. In a pioneering study, Kheirandish-Gozal et al. (2010) reported that not all pediatric OSA patients exhibited endothelial dysfunction, and that the number of EPCs was inversely related to the magnitude of endothelial dysfunction in each patient. More specifically, those children with OSA who presented endothelial dysfunction also presented lower EPCs values. In contrast, patients with no endothelial dysfunction had increased levels of EPCs and of the EPC-recruiting and homing chemokine stromal-derived factor-1α (SDF-1α) in blood when compared to controls. These findings in children add support to the notion that EPCs could be an essential mechanism for endothelial repair in OSA (Kheirandish-Gozal et al., 2010).

However, the mechanisms involved in EPCs recruitment, homing, and repair in OSA are not fully known. IH could have a potential role in EPCs mobilization through the stabilization and up-regulation of hypoxia-inducible genes, which in turn would increase plasma levels of VEGF (Lavie et al., 2002) and SDF-1α. These molecules are well-known for their intrinsic effect on EPCs recruitment and homing to the damaged endothelium. In this context, VEGF plasma levels are increased in OSA patients (de la Peña et al., 2008; Gozal et al., 2008). However, SDF-1α levels in patients could depend on the presence of endothelial dysfunction (Kheirandish-Gozal et al., 2010). Given that EPCs can repair injured vessels by homing to the sites of ischemia and neovascularization, it has been suggested that EPCs could play a cardioprotective role in OSA patients, particularly in cases of acute myocardial infarction (Berger and Lavie, 2011).

## Very Small Embryonic-Like Stem Cells in OSA

The recently described very small embryonic-like stem cells (VSELs) are pluripotent cells found in the bone marrow and peripheral blood of adult humans, and also in cord blood (Wojakowski et al., 2009). These cells, which have a small diameter of 3–6 μm, express markers of embryonic lineage, and are able to differentiate into lineage-committed cells from the three germ layers. It has been reported that VSELs are recruited from the bone marrow to the peripheral blood in several human pathologies: Crohn’s disease (Marlicz et al., 2012), stroke (Paczkowska et al., 2009), and acute myocardial infarction (Zuba-Surma et al., 2008; Wojakowski et al., 2009). There is also evidence that this population of stem cells can migrate in a gradient-dependent manner in response to cardiac developmental chemo-attractants such as SDF-1α, leukemia inhibitory factor (LIF), and hepatocyte growth factor (HGF) (Paczkowska et al., 2009; Gharib et al., 2010, 2011). The transplantation of this type of adult stem cells improved left ventricular function and myocyte hypertrophy induced in a model of myocardial infarction (Dawn et al., 2008). It has been suggested that the action mechanisms of VSELs could be both multilineage differentiation and paracrine effects on cardiac stem cells (Zuba-Surma et al., 2011).

The few data available on the potential role played by VSELs in OSA have been recently obtained in mice subjected to IH (Gharib et al., 2010) and continuous hypoxia (Gharib et al., 2011; Table 1). Animals subjected to IH consisting of cyclical exposures to either 21% O_2_ or 5.7% O_2_ breathing air every 3 min for 12 h/day (during the light period) for 2 days, mobilized VSELs from bone marrow into peripheral blood, as evidenced by a sixfold increase in the blood/bone marrow ratio of VSELs in those animals subjected to IH, compared to normoxic controls. A principal-component analysis of the transcriptome of VSELs showed segregation clustering into two groups associated with IH and normoxia, indicating a distinct genome-wide transcriptional perturbation in these pluripotent stem cells. In a second study by the same team, similar results were obtained in mice subjected to continuous hypoxia (8% O_2_) for 24 h. Furthermore, both studies also showed increased plasma levels of SDF-1α, HGF, and LIF, which could partly explain the recruitment of VSELs from bone marrow to peripheral blood in response to continuous and IH (Gharib et al., 2010, 2011).

## Mesenchymal Stem Cells in OSA

Mesenchymal stem cells (MSCs) are a heterogeneous subset of stromal stem cells that are present in many adult tissues. MSCs can differentiate into cells from the mesodermal lineage (adipocytes, osteocytes, and chondrocytes), as well as into cells from other embryonic lineages. MSCs can interact with immune cells by modulating several effector functions, and participate in organ homeostasis, wound healing, and slow aging processes (Williams and Hare, 2011). MSCs can migrate to injured tissues and have immunosuppressive effects (Semedo et al., 2009; Hoogduijn et al., 2010). Interestingly, it has been demonstrated that chronic hypoxia mobilizes MSCs from bone marrow into peripheral blood in rats (Rochefort et al., 2006) and their preconditioning to hypoxia can enhance their survival (Wei et al., 2012). Although the mechanisms involved in their mobilization from bone marrow are not known in detail, there is increasing evidence that chemokines (Ji et al., 2004; Abarbanell et al., 2009; Carreras et al., 2009) and hypoxia-inducible genes, including hypoxia-inducible factor-1α and VEGF (Liu et al., 2011), can stimulate the migration of MSCs to injured tissues and/or alter their function.

The potential role of MSCs in OSA has been studied recently using an acute rat model of recurrent airway obstructions mimicking OSA (Carreras et al., 2009, 2010a,b; Table 1). In the initial study, the authors reported a threefold increase in the number of CFUs-F from circulating blood of rats subjected to recurrent obstructive apneas (15 s apnea/min for 3 h), as compared to the number observed in control animals under normoxia (Carreras et al., 2009). Therefore, as in the case of other diseases and/or injurious challenges (Hoogduijn et al., 2010), this study suggested that MSCs may act as a modulator of inflammation, and could also repair the tissue damage induced by OSA.

A second study carried out with the same animal model of OSA showed that serum from rats subjected to recurrent obstructive apneas can activate MSCs (Carreras et al., 2010b). Specifically, serum from apneic rats was able to increase the motility, adherence, and reparative capacity of MSCs *in vitro*. A transwell setting was used to assess the chemotaxis of these cells. The application of serum from animals subjected to apneas in the lower compartment produced a twofold increase in the migration of MSCs. These findings indicate that recurrent obstructive apneas induce an early release of chemotactic proteins into the bloodstream that can, in turn, induce the mobilization of MSCs toward the affected tissues. Accordingly, the MSCs displayed greater adhesion to a monolayer of cultured endothelial cells when incubated in serum from rats subjected to recurrent obstructive apneas. An *in vitro* wound healing assay revealed that endothelial wound closure (repair of the denuded area) was greater in those endothelial cells cultured with serum from apneic rats.

Moreover, using an acute animal model of OSA, Carreras et al. (2010a) reported that MSCs had similar anti-inflammatory effects to those observed for myocardial infarction (Williams and Hare, 2011), acute kidney injury (Semedo et al., 2009), colitis (Gonzalez et al., 2009), and rheumatoid arthritis (Gonzalez-Rey et al., 2010). Moreover, the intravenous injection of MSCs immediately before the application of recurrent airway obstructions markedly reduced the early systemic inflammatory response triggered by apneas (Carreras et al., 2010a).

## Conclusion

The relatively limited number of currently available studies on the potential role played by adult stem cells in OSA suggests that these cells could play a role as a protective and reparative homeostatic response in this sleep breathing disorder, particularly with respect to its cardiovascular consequences. However, this research approach is very new, and available data are still scarce, particularly in the case of VSELs and MSCs. Whereas the data on EPCs in OSA are exclusively derived from patient studies, the data on VSELs and MSCs were obtained only in animal models. Moreover, the potential interactions between the usual OSA co-morbidities that are associated with cardiovascular risk (e.g., obesity, hypertension, diabetes) and their effects on the protective and reparative mechanisms of bone marrow-derived stem cells should be also taken into account in future research into this promising field. Specifically, a better characterization of the circulating levels of different adult stem cell types before and after CPAP treatment could help in defining different OSA phenotypes. This information could be useful to predict the relative risk of an individual patient to suffer cardiovascular consequences caused by OSA. Moreover, a further step of research should provide insights into the potential use of cell therapies to prevent or reduce the morbidity and mortality induced by this sleep breathing disorder.

## Conflict of Interest Statement

The authors declare that the research was conducted in the absence of any commercial or financial relationships that could be construed as a potential conflict of interest.
